# Efficient removal of allicin from the stalk of *Allium fistulosum* for dietary fiber production

**DOI:** 10.1038/s41538-024-00275-w

**Published:** 2024-06-14

**Authors:** Ye Li, Jiayin Ma, Yubin Cao, Dong Yang

**Affiliations:** 1 Beijing Key Laboratory of Functional Food from Plant Resources, College of Food Science & Nutritional Engineering, China Agricultural University, 17 East Tsinghua Rd., Beijing, 100083 China; 2 Jiangsu QingGu Foods Co., Ltd, Xingdong Economic Development Zone, Xinghua, 225700 China

**Keywords:** Biophysical chemistry, Process chemistry

## Abstract

The stalk of *Allium fistulosum* contains dietary fibers with complicated monosaccharide composition and glycosidic bond linkages, which renders it a better dietary fiber supplement. However, the unfavorable odor, majorly contributed by allicin, limits its applications. Although many physical and chemical methods have been developed to remove allicin, there is currently no comparison between their efficiencies. Here, we comprehensively compare all these methods of eliminating allicin in the *Allium* stalk by starting with optimization of the allicin extraction method. Results indicate that incubation of the chopped *Allium* stalk with water for 20 min and extraction with 75% ethanol reached a maximal extraction yield. Different methods of allicin elimination are examined, and physical removal of allicin by blanching at 100 °C reaches a maximal clearance rate of 73.3%, rendering it the most efficient and effective method eliminating allicin from the stalk of *Allium fistulosum* for the preparation of a totally green dietary fiber.

## Introduction

*Allium fistulosum*, also known as green onion, is a commonly seen vegetable and seasoning on the dining table worldwide since ancient times^1^. Polyphenols in *Allium fistulosum* exhibit antioxidant activity^2^, while ethanol and water extracts from *Allium fistulosum* could attenuate high-fat-diet induced obesity^3^. The stalk part contains about 16% polysaccharides with complicated monosaccharide composition and glycosidic linkages^1,4^, and it is proven that dietary fibers with more complicated chemical structures could support microbiota growth in the gut with higher richness^4^. Thus, the stalk of *Allium fistulosum* is of great nutritional value to develop functional foods and dietary supplements.

Although allicin exhibits multiple health beneficial effects, including anti-oxidative, pathogen resisting, gut flora regulating, and cancer inhibiting effects^5,6^, however, its characteristic odor renders *Allium* vegetables an unfavorable choice by certain groups of consumers as allicin is produced in chopped or crushed *Allium* to defend themselves from the attack by pathogens and pests^7^. Dietary fiber extracted from *Allium* usually exhibits the unpleasant odor from allicin which prevents its wide application as a dietary fiber supplement. As a matter of fact, elimination of allicin has been widely studied previously. Thermal decomposition of allicin shows a first-order reaction kinetics^8^. On the other hand, efficient blanching of frozen *Allium fistulosum* retained most of its allicin content^9^. Life experience indicates that drinking tea could reduce the allicin odor of so called “garlic breath”, suggesting tea polyphenols could also possibly eliminate allicin^10^. Additionally, citric acid seems to increase allicin content in treated garlic (*Allium sativum* L.) compared to dried slices^11^. However, the detailed impact of citric acid on the allicin content of *Allium fistulosum* remains unknown.

To eliminate the unpleasant odor and make the stalk of *Allium fistulosum* a more acceptable dietary fiber supplement, efficient and economical removal method(s) of allicin should be developed. Here, we comprehensively compared almost all the currently developed methods of allicin removal in *Allium fistulosum*, such as physical treatment of freezing and blanching, chemical treatment including tea polyphenols and citric acid, and their efficiency to offer a reference for the production of dietary fiber supplement from the stalk of *Allium fistulosum*.

## Results and discussion

### Optimization of allicin determination in the stalk of *Allium fistulosum*

Although allicin related studies have been performed for more than thirty years, determination of the maximal amount of allicin in *Allium* has not been optimized^12^. Accurate determination of allicin was previously developed by HPLC coupled with a UV detector at 242 nm, however, such expensive instrumentation renders this method far away from being simple^13^. Most of the allicin determination methods were based on its reaction with cysteine, and the remaining cysteine was then determined with DNTB^14^. Even in the original method determining allicin content from garlic powder, detailed method of extracting allicin from *Allium* was not optimized^14^.

**Fig. 1:**
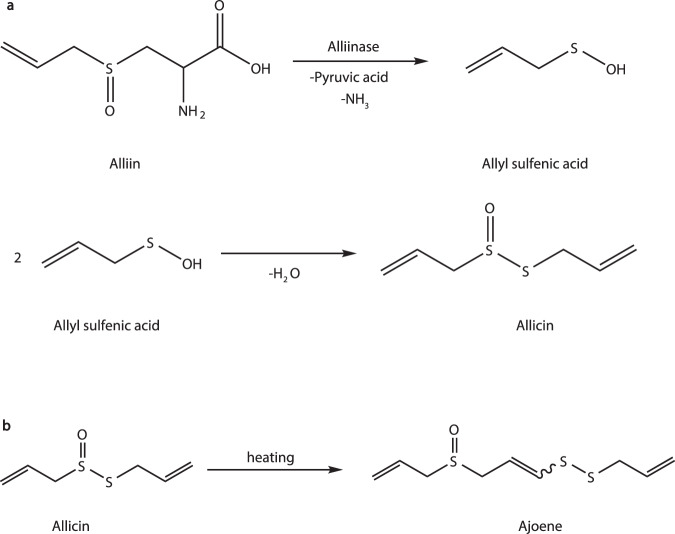
Allicin generation and its thermal degradation. **a** Alliin in *Allium* exposed to alliinase transformed into allyl sulfenic acid, and the latter transformed into allicin via dehydration condensation. **b** Allicin at high temperatures was transformed into ajoene.

**Fig. 2:**
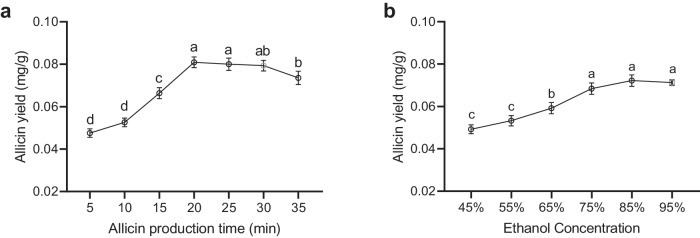
Optimization of allicin determination in the stalk of *Allium fistulosum.* **a** Allicin extraction yield change as production time of allicin upon the mixing of water with chopped stalk elongates. **b** Allicin extraction yield change as ethanol concentration used in the extraction of allicin produced from chopped stalk increases. Different letters indicate difference at *p* < 0.1 and error bars represent standard deviations.

There are majorly two steps of allicin extraction from *Allium*, incubation with water and extraction with ethanol. The first step is to extract allicin produced by alliinase catalyzed alliin transformation in broken *Allium* tissues (Fig. 1a)^15^. Two factors involved, the incubation time and ethanol concentration, were optimized in this study. Firstly, the incubation time of chopped *Allium fistulosum* in water was examined from 5 to 35 min at 26 °C. In the following allicin determination, it was shown that the extraction yield increased significantly as incubation prolonged from 5 min till 20 min (Fig. 2a). Further elongation of incubation time did not increase the extraction yield of allicin. Additionally, incubation of *Allium fistulosum* in water for 35 min even decreased the allicin extraction yield, this is probably due to the volatile nature of allicin.

Secondly, for the ethanol concentration during the extraction step, the concentration of 45%-95% ethanol was examined. It was shown that as the concentration of ethanol increased from 45% to 75%, allicin extraction yield increased significantly (Fig. 2b). Further increase of ethanol concentration from 75% to 95% did not increase the extraction yield. Thus, in the following experiments where allicin was determined, the incubation time of chopped *Allium fistulosum* in water was subjected to 20 min incubation and the ethanol concentration of 75% was used to maximally extract allicin.

### Chemical removal of allicin in the stalk of *Allium fistulosum*

It is generally believed that rinsing mouth with tea can help improving oral health^16,17^, and additionally eliminating the unpleasant odor after eating raw garlic or other *allium* containing foods. It is thus possible that some tea content reacts with allicin, and highly likely this content is tea polyphenols since it can react with many food ingredients^18^. Based on the fact that addition of tea polyphenols into food supplements brings about antioxidative properties and improvement on physical properties^19,20^, here the effect of tea polyphenols on the clearance of allicin in the stalk of *Allium fistulosum* is examined.

**Fig. 3:**
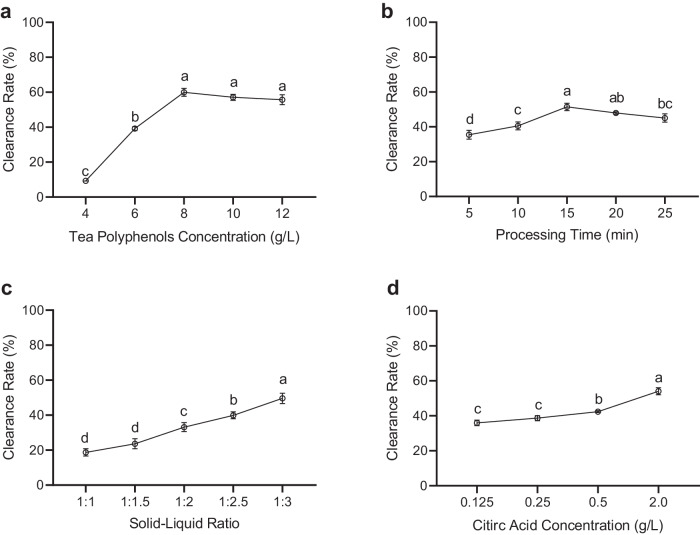
Chemical removal of allicin in the stalk of *Allium fistulosum.* **a** Effect of tea polyphenols concentration on the removal of allicin in chopped *Allium* stalk via incubating with tea polyphenols. **b** Effect of processing time on the removal of allicin in chopped *Allium* stalk via incubating with tea polyphenols. **c** Effect of solid to liquid ratio on the removal of allicin in chopped *Allium* stalk via incubating with tea polyphenols. **d** Effect of citric acid concentration on the removal of allicin in chopped *Allium* stalk via incubating with citric acid. Different letters indicate difference at *p* < 0.1 and error bars represent standard deviations.

Tea polyphenols solution was added to the chopped *Allium fistulosum* so that allicin produced could be eliminated. Firstly, tea polyphenols concentration was optimized by determining residual allicin after treatment with 4-12 g/L tea polyphenols. It was shown that as tea polyphenols concentration increased from 4 to 8 g/L, the clearance rate increased significantly, and further increase of tea polyphenols concentration did not improve the clearance rate (Fig. 3a). Secondly, tea polyphenols processing time was optimized by determining residual allicin after treatment for 5-25 min. It was shown that the clearance rate increased significantly when the processing time prolonged to 15 min, however, it did not continue to increase after 15 min (Fig. 3b). Thirdly, the solid-liquid ratio in the tea polyphenols treatment was optimized, and it was shown that the clearance rate increased significantly when the solid-liquid ratio increased from 1:1.5–1:3 (Fig. 3c).

OPLS-DA was employed to analyze the weight of each factor, including processing time, tea polyphenols concentrations, and solid-liquid ratios on their impact of allicin removal during tea polyphenols treatment (Supplementary Table 1). A combination of 9 sets of experiments were performed, and the influence of the factors were ranked as processing time>polyphenols concentration>solid-liquid ratio (Table 1). The best combination of experimental set up is processing time of 20 min, polyphenols concentration of 6 mg/mL, and solid-liquid ratio of 1:1.5. Under these conditions the experiment was performed in triplicates, and the average clearance rate of allicin was 51.43%.

**Table 1:** Results of orthogonal optimization

Trial	Processing Time	Solid-liquid ratio	Concentration	Clearance rate
1	1	1	1	0.2259
2	2	2	2	0.2499
3	3	3	3	0.2918
4	1	2	3	0.2775
5	2	3	1	0.3253
6	3	1	2	0.2794
7	1	3	2	0.0302
8	2	1	3	0.2554
9	3	2	1	0.5109
K1	0.534	0.761	1.062	
K2	0.831	1.038	0.560	
K3	1.082	0.647	0.825	
K1	0.178	0.254	0.354	
K2	0.277	0.346	0.187	
K3	0.361	0.216	0.275	
R	0.183	0.130	0.168	
Optimal level	A > C > B			
Optimal combination	A3C1B2			

Based on the above clearance effect of tea polyphenols and its weak acidic character, the effect of acidic reagents on allicin clearance becomes worthy to investigate. Citric acid is a strong, organic, natural acid that has been classified as generally recognized as safe by the U.S. Food and Drug Administration, and it can serve as a food additive for antioxidative and aflatoxin clearance purposes^21,22^. However, whether citric acid could interact with allicin in *Allium* remains largely unknown.

Citric acid solution was added to the chopped *Allium fistulosum* to test whether allicin could be eliminated. Citric acid concentration was optimized by determining residual allicin after treatment with 0.125–2.0 g/L citric acid. It was shown that as citric acid concentration increased from 0.125 to 0.25 g/L, the clearance rate did not increase significantly, however, further increase of citric acid concentration from 0.25 to 2.0 g/L did significantly improve the clearance rate (Fig. 3d). The maximal clearance rate with 2.0 g/L citric acid was 54%.

### Physical removal of allicin in the stalk of *Allium fistulosum*

**Fig. 4:**
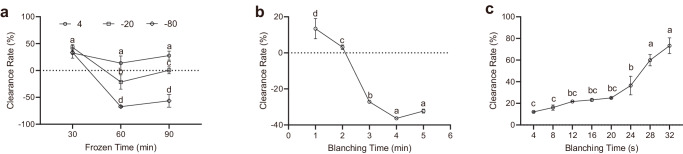
Physical removal of allicin in the stalk of *Allium fistulosum.* **a** Effect of temperature and frozen time on the removal of allicin in chopped *Allium* stalk. **b** Effect of blanching time at 60 °C on the removal of allicin in chopped *Allium* stalk. **c** Effect of blanching time at 100 °C on the removal of allicin in chopped *Allium* stalk. Different letters indicate difference at *p* < 0.1 and error bars represent standard deviations.

It was reported that freezing impact the sensory properties and bioactive compounds of *Allium ampeloprasum* Var. bulga^23^, *Allium ampeloprasum* Var. porrum^24^, *Allium cepa* L.^25^, and black garlic^26,27^. However, the effect of freezing on the stalk of *Allium fistulosum*, especially its allicin content, remains unknown. To remove allicin content by freezing, its content was examined at three temperatures, 4 °C, −20 °C, and −80 °C, for different time scale from 0.5 hour to 1.5 hours. It was shown that at all temperatures, the clearance rate of freezing treatment for half an hour was around 38% (Fig. 4a). As the freezing time prolonged to one hour, the clearance rate decreased, and even to negative values at −20 °C, and −80 °C. When freezing continued till one and half hours, the clearance rate slightly increased, but not to an extent of these treated for half an hour.

It was reported that thermal treatment, specifically blanching, could impact the allicin content in *Allium sativum* L. in the slice, paste, and fresh garlic form^28–30^. The chemistry behind is that allicin underwent thermal transformation into a larger molecule of ajoene (Fig. 1b)^31^. However, the effect of blanching on the allicin content of *Allium fistulosum* stalk remains unknown. Hence, the allicin content was examined at the blanching temperature of 60 °C first. After treatment of 1 min, the clearance rate was 13.4% (Fig. 4b). As the treatment time prolonged, the clearance rate decreased to 3.2% at 2 min, and then to negative values at 3 min. The clearance rate reached a negative maximal of -36.3% at treatment of 4 min. On the other hand, blanching at 100 °C for 4 sec reached a clearance rate of 12.1%, and prolonged blanching slightly increased the clearance rate till 25% at 20 sec (Fig. 4c). Further prolongation of blanching time significantly increased the clearance rate till 73.3% at 32 sec.

Here in this study, the extraction condition of allicin from the chopped stalk of *Allium fistulosum* was optimized to be incubation with water for 20 min and extraction with 75% ethanol. Chemical elimination of allicin with tea polyphenols reached a maximal clearance rate of 51.4% and with citric acid reached a maximal clearance rate of 54%. Physical removal of allicin by freezing reached a maximal clearance rate of 43%, by blanching at 60 °C reached a maximal clearance rate of 13%, and by blanching at 100 °C reached a maximal clearance rate of 73.3%. In conclusion, blanching at high temperatures is the most efficient and effective among all methods eliminating allicin from the chopped stalk of *Allium fistulosum*.

## Methods

### Materials

*Allium fistulosum* used in this study was cultivated in Shandong Province, China, and the stalk parts were obtained by cutting off the green parts. The stalk part was further processed as described in the following section. Absolute ethanol, cystine, 5,5’-dithio-bis (2-nitrobenzoic acid) (DNTB), and citric acid of analytical grade was purchased from Sinopharm Chemical Reagent Co., Ltd., Shanghai, China. Tea polyphenols were purchased from Red Star pharmaceutical, Anhui Province, China.

### Allicin determination

Chopped *Allium fistulosum* stalk of around 5 g was added into 5 mL of water and incubated at 26 °C for 15 min before extraction with ethanol. The extraction was performed at 26 °C for 50 min, centrifuged at 6000 rpm/min for 10 min, and the supernatant was subjected to the following measurement. Optimization of allicin extraction as performed by varying the incubation time with water, and ethanol concentration.

Determination of allicin from differently treated *Allium* stalk was basically the same as previously described with modifications^12^. Principally, allicin extracts were added into a HEPES (50 mM) buffer containing 1 mM cystine and 2 mM DNTB, allowed to react in dark for 15 min, and the absorbance at 412 nm was measured with a DeNovix DS-11 FX + spectrophotometer (DeNovix Inc, Wilmington DE, USA).

### Chemical removal of allicin in the stalk of *Allium*

Removal of allicin in the stalk by tea polyphenols. Effect of tea polyphenol concentration on the removal of allicin was determined as the following. A series of concentrations of tea polyphenols water solutions, including 4 mg/mL, 6 mg/mL, 8 mg/mL, 10 mg/mL, and 12 mg/mL were added into the chopped *Allium* stalk with a solid-liquid ratio of 1:3, respectively. The mixture was incubated for 5 min at 25 °C before the tea polyphenols solution was discarded. Residual allicin was extracted and determined the same as described previously.

Effect of processing time on the removal of allicin was determined as the following. Tea polyphenols solution of 6 mg/mL was added into the chopped *Allium* stalk with a solid-liquid ratio of 1:3, respectively. The mixtures were incubated for 5 min, 10 min, 15 min, 20 min, and 25 min, respectively, before the tea polyphenols solution was discarded. Residual allicin was extracted and determined the same as described previously.

Effect of solid-liquid ratio on the removal of allicin was determined as the following. Tea polyphenols solution of 6 mg/mL was added into the chopped *Allium* stalk with a solid-liquid ratio of 1:1, 1:1.5, 1:2, 1:2.5, and 1:3, respectively. The mixtures were incubated for 5 min before the tea polyphenols solution was discarded. Residual allicin was extracted and determined the same as described previously.

Orthogonal partial least squares discriminant analysis (OPLS-DA) was developed by applying an orthogonal projection to the PLS-DA algorithm to construct the variations of the predictors correlated but orthogonal to the response^32^. Thus, it was performed here to select important variables in the tea polyphenols treatment of allicin and designed by SSPSAU (QingSi Technology Ltd., Beijing, China). Tea polyphenols treatment time, solid-liquid ratio, and tea polyphenols concentration were examined in the test with three levels. Treatment time of 10 min, 15 min, and 20 min, solid-liquid ratio of 1:1, 1:1.5, and 1:2, tea polyphenols concentration of 6 mg/mL, 8 mg/mL, and 10 mg/mL were used in the experimental setup (Supplementary Table 1).

Removal of allicin in the stalk by citric acid was performed as the following. A series of concentrations of citric acid water solution, including 0.125 mg/mL, 0.25 mg/mL, 0.5 mg/mL, 1.0 mg/mL, and 2.0 mg/mL were added into the chopped *Allium* stalk respectively. The mixtures were incubated for 5 min before the citric acid solution was discarded. Residual allicin was extracted and determined the same as described previously.

### Physical removal of allicin in the stalk of *Allium*

Frozen treatment of allicin removal in the stalk of *Allium* was performed as the following. The chopped *Allium* stalk was chilled at 4 °C, −20 °C, and −80 °C for 0.5 h, 1.0 h, and 1.5 h before allicin extraction and determination as described aforementioned, respectively.

Blanching treatment of allicin removal in the stalk of *Allium* was performed as the following. The chopped *Allium* stalk was blanched at 60°C for 1 min, 2 min, 3 min, 4 min, and 5 min, respectively. In the other blanching treatment, the temperature was set at 100 °C and the treatment time was set for 4 sec, 8 sec, 12 sec, 16 sec, 20 sec, 24 sec, 28 sec, and 32 sec, respectively. Allicin extraction and determination were then performed as described previously.

### Statistical analysis

All results were plotted with Prism (v 8.0, GraphPad Software Inc, La Jolla CA, USA). One-way ANOVA was applied to the data with the Duncan test for significance analysis using SPSS Statistics (v 17, IBM Corp., Armonk, NY, USA).

### Reporting summary

Further information on research design is available in the Nature Research Reporting Summary linked to this article.

### Supplementary information

Supplementary information

reporting summary

## Data Availability

The authors declare that data supporting the findings of this study are available within the paper and its supplementary information files.
