# Supplementary material for: Efficient removal of allicin from the stalk of Allium fistulosum for dietary fiber production
Source: NPJ Sci Food. 2024 Jun 14;8:32. doi: 10.1038/s41538-024-00275-w (PMC11178807; doi:10.1038/s41538-024-00275-w)
Supplement: Supplementary file 1 — Supplementary information [file 41538_2024_275_MOESM1_ESM.pdf]

Supplementary information

Efficient removal of allicin from the stalk of Allium fistulosum for dietary fiber production

Ye Li[1,2], Jiayin Ma[1,2], Yubin Cao[3], and Dong Yang[1,*]

[1]Beijing Key Laboratory of Functional Food from Plant Resources, College of Food Science & Nutritional Engineering, China Agricultural University, 17 East Tsinghua Rd., Beijing 100083, China

[2]These authors contributed equally to this work.

[3]Jiangsu QingGu Foods Co., Ltd, Xingdong Economic Development Zone, Xinghua 225700, China

Supplementary table 1 Orthogonal test factors and levels

Level	Factors		
	A Treatment time (min)	B Solid-liquid ratio	C Concentration (g/L)
1	10	1:1	6
2	15	1:1.5	8
3	20	1:2	10
